# Adjuvant-Induced Arthritis in Guinea Pigs

**Published:** 2016

**Authors:** O.S. Taranov, S.N. Yakubitskiy, T.S. Nepomnyashchikh, A.E. Nesterov, S.N. Shchelkunov

**Affiliations:** State Research Center of Virology and Biotechnology VECTOR, Kol’tsovo, Novosibirsk region, 630559 Russia

**Keywords:** rheumatoid arthritis, adjuvant-induced arthritis, guinea pig

## Abstract

We propose a model of rheumatoid arthritis (RA) induced in outbred guinea pigs using a single subcutaneous injection of complete Freund’s adjuvant to the hind paw. Histological examination of this model shows fibrin deposition on the surface of the synovial membrane, leukocyte infiltration of the synovial membrane and adjacent tissues, proliferation of the granulation tissue, and emergence of angioid areas, characteristic of RA. The cell response appears as an increase in the plasma cell count and development of follicle-like lymphoid infiltrates; erosion of the articular surface of the cartilage, frequently with deep cartilage destruction over large areas; and epiphysiopathy. The high reproducibility of arthritis induction in this RA model has been demonstrated. The proposed model is promising for the assessment of anti-arthritis preparations and dosage regimens.

## INTRODUCTION

Rheumatoid arthritis (RA) is a heterogeneous autoimmune disease characterized by a systemic inflammation of the connective tissue. The morphological presentation of RA includes the inflammation of the articular capsule tissue, proliferation and hyperplasia of synovial cells, pannus formation, and structural destruction of cartilage and the subchondral bone. RA is characterized by a steadily progressive disease of the joints and internal organs, leading to early disability and shortened life expectancy. About 70 million people worldwide suffer from RA. Most often, the disease attacks older people. The reasons that cause the development of arthritis remain not well established, possibly because of their multiplicity [1, 2 , 3, 4].

Various animal models are used to study the pathogenesis of RA and develop drugs effective against the disease. The vast majority of research on the induction and therapy of RA are performed on certain lines of mice and rats [1, 2, 5, 6]. This is first of all due to the good state of knowledge on and homogeneity of these animals and the large number of available immunological reagents and test systems. However, none of these models fully reflects all aspects of the development of human RA [1, 5, 7] and, therefore, preclinical studies of potential antiarthritic drugs should be carried out in various animal models.

The genetic heterogeneity of humans and probable multiplicity of arthritis induction mechanisms actualize the use of laboratory animals that are close to humans in their physiological and immunological properties as RA models. It is believed that guinea pigs are more appropriate models on which to study and develop treatments for various human inflammatory and infectious diseases than mice and rats [8-10].

Our work was aimed at developing a reproducible model of adjuvant-induced arthritis (AIA) in outbred guinea pigs.

## EXPERIMENTAL

**Animals**

We used female outbred guinea pigs weighing 200–250 g (3–4 weeks of age) received from the animal kennel of the State Research Center of Virology and Biotechnology *VECTOR*. The animals were kept under natural light conditions with constant access to water and food.

**Arthritis induction using Freund’s adjuvant**

The animals were subcutaneously injected with a Freund’s complete adjuvant (CFA) solution **in**to the hind paw at a dose of 50, 100, or 200 μm, which corresponded to groups 50, 100, and 200, respectively. The control group guinea pigs (C) were administered 200 μl of a sodium phosphate buffer (PBS). Each of the control group and group 50 consisted of 9 guinea pigs; group 100 – 14; and group 200 – 10 animals.

The width of the distal metatarsal region of both hind paws was measured one day after CFA injection and then every 3–4 days. Group C, 50, and 200 animals were taken out of the experiment (2–3 animals at a time), using ether anesthesia according to the animal use regulations in compliance with humanity principles as stated in the European Community Directives (86/609/EEC) and Helsinki Declaration, on the 16, 28, 42, and 56th day. CFA-induced pathological changes were assessed based on a histological examination of tissues of both hind limbs of the experimental and control animals. All group 100 animals were taken out of the experiment on day 28th after CFA injection.

**Histological analysis**

The hind limbs of the guinea pigs were fixed in a 10% neutral formalin solution for histological examination (BioVitrum, Russia) for 48 hours. Then, bone-containing samples were decalcified using a BioDec R preparation (Bio Optica Milano, Italy) for 48–72 hours. After decalcification, the animals’ hind paws were dissected in the sagittal direction as described in [2] in order to prepare histological specimens. Further material processing was performed according to the standard procedure for histological preparations: sequentially dehydrated in alcohols with increasing concentrations, soaked in a xyloparaffin mixture, and embedded in paraffin blocks. Paraffin sections (4–5 μm thick) were prepared using an automatic rotary microtome HM 360 (Germany). Sections were stained with hematoxylin and eosin. Differential staining of connective tissue was carried out using the Van Gieson’s stain. Light optical examination and microphotography was performed on a AxioImager Z1 microscope (Carl Zeiss, Germany) equipped with a high resolution digital camera HRc (Carl Zeiss, Germany), using the AxioVision 4.8.2 software package (Carl Zeiss, Germany).

The statistical analysis of the results of the histological analysis of the samples obtained from group 100 animals was done using a point-based assessment of the severity of pathological manifestations of the disease in different groups. The disease severity was assessed based on four criteria: inflammatory response in articular capsule tissues, severity of synovitis (pannus formation), cartilage erosion, and destruction of the articular surface of the bone.

The inflammatory response was scored as 1 point in the case of periarticular tissue infiltration with isolated lmphocytes and granulocytes; 2 points – in the case of significant infiltration, formation of small lymphocytic-histiocytic nodules, and moderate swelling of the periarticular tissue. In the case of severe diffuse soft tissue infiltration with macrophage cells, lymphocytes, granulocytes, and plasma cells, formation of a dense infiltration, and significant edema, the inflammatory response was scored as 3 points.

The development of synovitis was assessed as follows: 1 point – there are signs of swelling of the synovial membrane and synoviocyte proliferation; 2 points – actively proliferating granulation tissue forms pannus at the points of contact between the synovium and cartilage, forming the articular surface; 3 points – pannus “overlaps” on the articular cartilage and destroys it.

Cartilage destruction was evaluated based on damage depth: 1 point – erosion of the surface layer (articular surface) or emergence of cell-free fields, 2 points – lesions spreading to the mid-thickness of the cartilage; 3 points – destruction involves the whole cartilage.

Severity of bone destruction: 1 point – there are small areas of resorption in the epiphyseal areas of the bones that form the join; 2 points – local injury to the peri-epiphyseal cortical layer; 3 points – extensive damage to the cortical layer, destruction of trabeculae of the cancellous bone, and bone deformity.

## RESULTS

**Clinical manifestations of arthritis in guinea pigs injected with CFA**

**Fig. 1:**
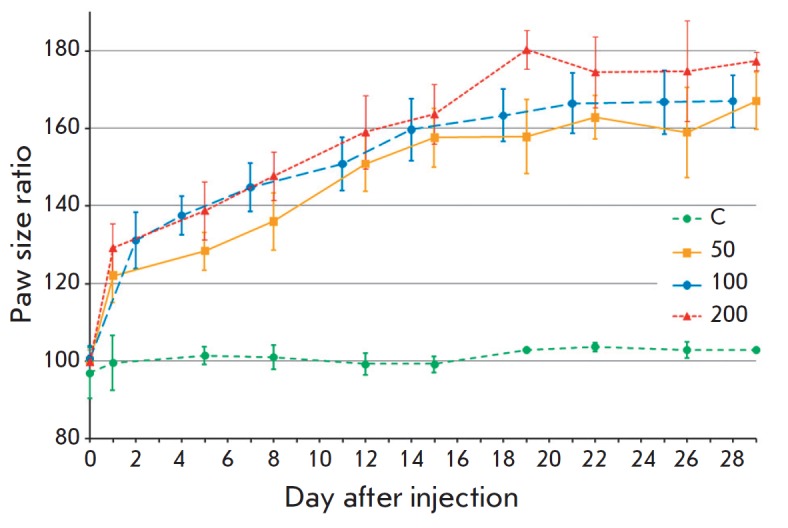
Development of inflammatory response in the left hind limb of guinea pigs after the injection of the complete Freund’s adjuvant at a dose of 50 (group 50), 100 (group 100), or 200 (group 200) μl. The percentage ratio of the width of the experimental left paw to that of the intact right paw is shown (as the arithmetic mean per group ± standard deviation); C, control animal group injected with 100 μl PBS into the left paw.

**Fig. 2:**
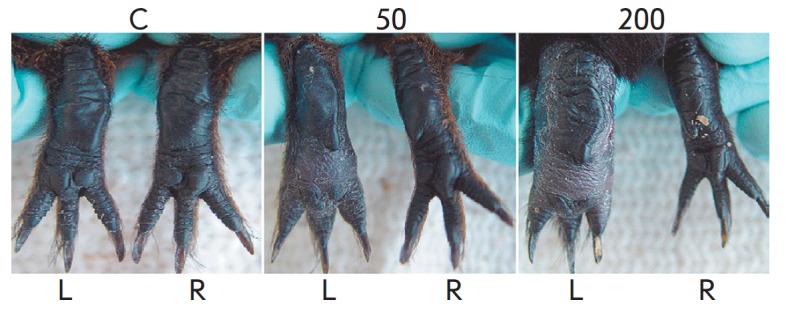
Appearance of the hind limbs of the control group (C), groups 50, and group 200 guinea pigs on day 37 after CFA injection; L and R stand for left and right paws.

Outbred guinea pigs were subcutaneously injected with CFA into the hind paw at a dose of 50, 100, or 200 μl. The width of the distal metatarsal area of both hind paws was measured, and it was found that the hind limb injected with adjuvant increased in size as a result of an inflammatory response *(Fig. 1)*. The response (swelling and inflammation) to the CFA injection manifested itself as early as on the first days after the injection, swelling of the treated paw rapidly increased during the first 2 weeks, and then this value almost reached a plateau. The most severe swelling, which covered almost the entire paw, occurred in group 200 *(Fig. 2)*.

**Histological analysis of the hind paws of AIA guinea pigs, group 200**

Pathomorphological symptoms of arthritis were similar in all groups of AIA animals. The most significant changes were observed in the left hind paw (on the side of the injection of the inducing CFA preparation) in the 200 group. For this reason, further discussion herein refers to this particular group.

**Fig. 3:**
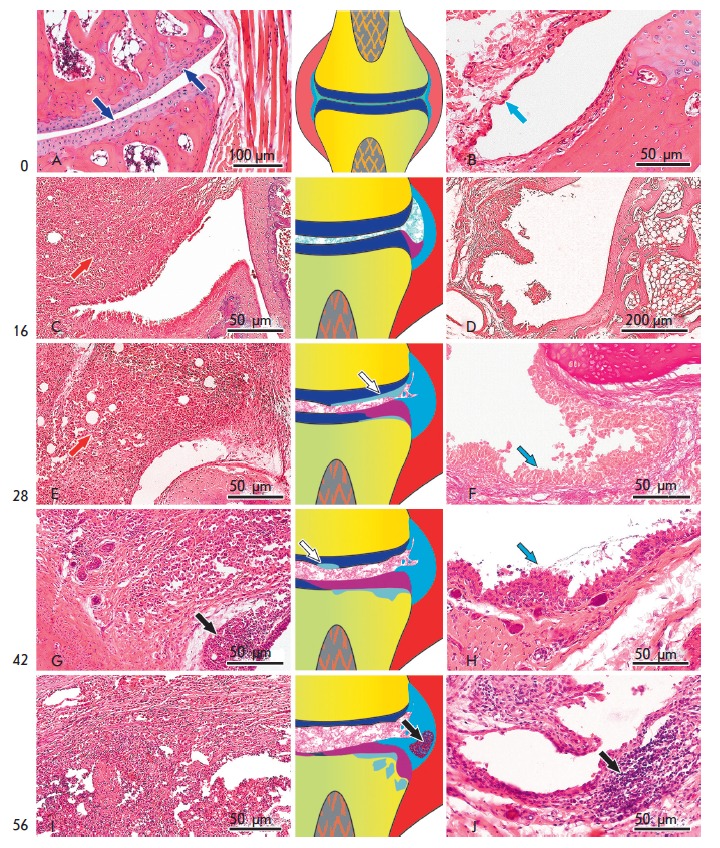
The dynamics of inflammatory signs in the periarticular tissue (left column) and synovial membrane (right column); see body text for details. Hematoxylin and eosin staining (F, staining according to Van Gizon). In the schemes, the main joint elements and the pathological signs of arthritis (central column) are color-coded: bone tissue, yellow; cartilage, blue; joint capsule (inflammation), red; synovial membrane, cyan; pannus, magenta; damaged bone trabeculae in the cancellous bone, gray. The corresponding structures are indicated with arrows in micro images; arrow colors corresponds to the colors in the scheme. White arrows show erosive lesions on the articular cartilage surface and black arrows show granuloma under the synovial membrane.

**Fig. 4:**
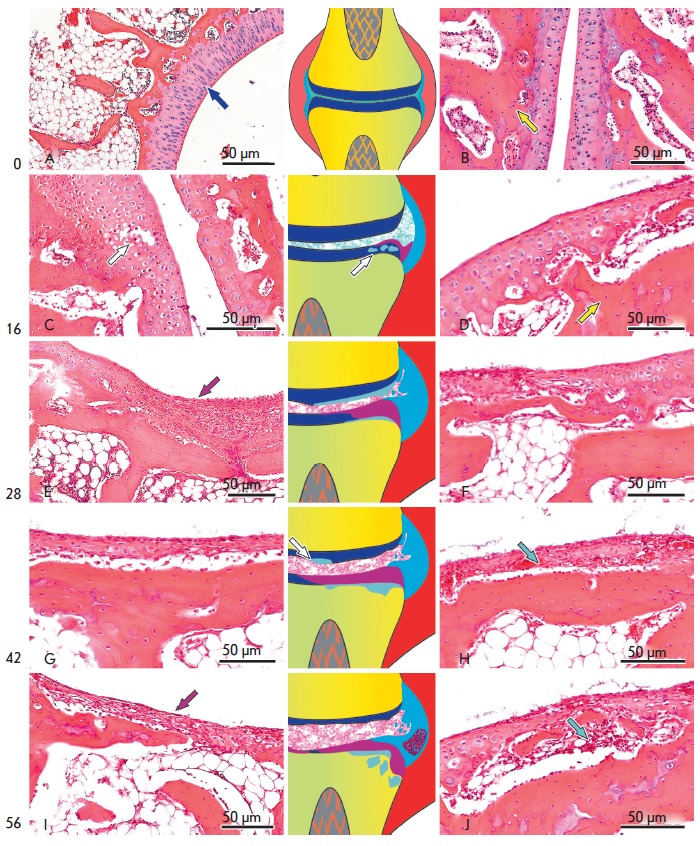
The dynamics of articular cartilage (left column) and underlying bone (right column) lesions; see text for details. Hematoxylin and eosin staining. The color coding in the schemes (central column) is the same as in Figure 3.

By day 16th, of the experiment, a diffuse dense cellular infiltration was observed in the reticular dermis, subcutaneous fat tissue, and between muscle fibers, while the surface layers of the skin remained intact. The infiltrate was dominated by granulocytes (mostly neutrophils), macrophages, and a small amount of plasma cells. Necrotic disintegration zones were sometimes observed in the central portion of large infiltration regions. Pronounced edema, inflammatory cell infiltration *(Fig. 3C)*, and hemodynamic disorders in the form of a significant plethora of small-and medium-caliber vessels, stasis, and thrombosis of small vessels of the venous bed were observed around the joints. The synovium was swollen and significantly thickened due to cell proliferation. In the marginal zone of the synovium, at the point of its contact with cartilage, development of granulation tissue and its spread to the articular surface of the cartilage was observed *(Fig. 3D)*. Cartilage lesions were limited to variously sized erosions, cavities, and formation of cell-free zones in the cartilage *(Fig. 4C)*. No changes in the subchondral bone tissue were observed at this stage of the experiment *(Fig. 4D)*.

By day 28, the severity of the edema and inflammatory cell infiltration in the periarticular soft tissues increased *(Fig. 3E)*. Joint disease progressed. The inflammatory process mainly involved large joints: ankle and talo-navicular ones. Small tarsus and metatarsus joints were damaged to a lesser degree. Dramatic thickening of the synovium covering the villi was observed, as well as proliferating synovial cells arranged in multiple layers. In some cases, the synovial cells exfoliated into the joint cavity *(Fig. 3F)*. In the subepithelial layer, the perivascular space was dilated, there was a pronounced perivascular edema, swelling and delamination of muscular coat of the vessels, swelling and thickening of the endothelium, and margination and adhesion of neutrophils on endothelial cells. Overgrowth of granulation tissue, which “overlapped” with the cartilage in the form of pannus, was observed along the margins of the articular cartilage *(Fig. 4E)*. There were varying degrees of cartilage lesions. Typically, rather large fields of surface layer erosion and destruction areas were observed, which sometimes extended over the whole thickness of the cartilage. Thinning of the subchondral bone plate and emergence of rather extensive zones of cortical layer destruction were observed *(Fig. 4F*).

42 days after the CFA injection, massive infiltration of the soft tissue of the foot with necrotic areas at the central portion of the infiltration was observed on the side of the injection. Angiomatosis, congestion, and a large number of fibroblasts were observed on the periphery of infiltration foci, indicating the beginning of the active repair process of articular capsule tissue *(Fig. 3G)*. The synovium was strongly edematous, and its hypertrophic villi deeply penetrated the articular cavity. An active process of vascular and connective tissue regeneration was observed under the synovium and in the villi *(Fig. 3H)*. There were multiple lesions of the cartilage, which involved both large joints and small metatarsal joints. The severity of the degradation also varied from marginal deformation of the cartilage *(Fig. 4G)* to a complete cartilage loss over the large area of the articular surface and local destruction of bone trabeculae of the cancellous bone *(Fig. 4H)*.

On day 56 of the experiment, a certain decrease in the activity of the inflammatory process was observed, along with progression of tissues sclerosis around the inflammatory infiltrate *(Fig. 3I)*. In some cases, the connective tissue grew into the infiltration, forming smaller cell fibrotic granulomas surrounded by a fibrous capsule. Despite the decrease in the inflammatory response in the surrounding tissues, inflammation of the synovium remained active. Various degrees of synovitis were observed in all animals. Along the diffuse infiltration, small granulomatous nodules were often found under the synovium *(Fig. 3J)*. Pronounced degradation of the articular cartilage *(Fig. 4I)* and granulation tissue growth into the cancellous bone was observed *(Fig. 4K)*.

In the right untreated hind limbs, inflammation manifested itself in the form of small foci of inflammatory cell infiltration, mainly along small-caliber blood vessels. However, synovitis symptoms were observed 42 and 56 days after the injection of adjuvant, although to a much lesser degree than on the side of the CFA injection. Moreover, erosive lesions of the articular cartilage in these extremities were observed in 56 days.

**Histological analysis of the hind limbs of guinea pigs with AIA, group 50.**

The pathological changes in the hind limbs of group 50 guinea pigs differed from those in group 200 mainly in their severity. In group 50, inflammation in the articular capsule and surrounding tissues attained a maximum 28 days after the CFA injection, while in group 200, the active inflammatory process continued as long as 42 days into the experiment. By day 28, the first signs of recovery around the inflammatory focus were observed in both groups in the form of vascular proliferation and proliferation of spongy irregular connective tissue. Deep cartilage erosion and destructive lesions of the cortical layer of the bone were observed in the joint, which worsened over time. However, the severity of these signs was significantly less pronounced compared to group 200. Moreover, the pathological process typically involved one joint, while in group 200, two or even three hind limb joints were involved.

**Reproducibility of AIA in guinea pigs, group 100**

All 14 guinea pigs in group 100, which was used to assess the reproducibility of the AIA, developed synovial and articular cartilage lesions on the side of the CFA injection (left hind paw) characteristic of rheumatoid arthritis. In addition to the cartilage surface erosion observed in all animals in this group, the destruction involved half the thickness of the articular cartilage in half of the cases. In two cases, there were portions of completely destroyed cartilage and the underlying bone was damaged. In four animals, articular cartilage of the ankle was not involved despite a rather significant inflammation in the articular capsule and synovium tissues. At the same time, in these animals, cartilage destruction was observed in other joints.

**Fig. 5:**
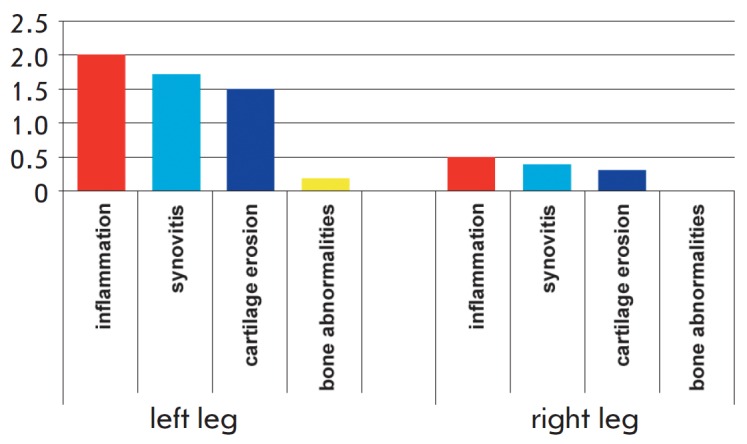
Comparative characteristics of the severity of the injury to the periarticular and articular tissues in the guinea pig hind limbs: left, on the side of the CFA injection; right, in the symmetrical intact limb. The mean values for group 100 are shown.

There was almost no inflammation in the limbs symmetrical to the CFA injection sites. There were only small foci of inflammatory cell infiltration along the small-caliber blood vessels. However, moderate manifestations of synovitis were observed in half of the animals, and erosive lesions of the articular cartilage were observed in two animals. Moreover, in one case, there was a limited area where cartilage tissue was destroyed over the full thickness of the articular cartilage, which, however, did not fit into the overall picture of lesions in this group of animals. The severity of various pathological manifestations in individual animals as assessed on the three-point scale (see. Experimental) is reported in the *Table*, and the average totals for the whole group 100 are shown in *Fig. 5*.

## DISCUSSION

It is known that RA is a disease characterized by a diversity of molecular mechanisms and the sensitivity of individuals to different therapeutic agents. Therefore, the use of different laboratory models is advisable when testing potential antiarthritic drugs [1, 2, 3, 5, 6, 11]. Currently, the most well studied induced arthritis models are based on certain lines of mice and rats [1, 2, 5, 6]. However, none of these models reflect all the characteristic features of RA in humans [5] and, therefore, the search for additional experimental models of RA remains relevant.

We evaluated the effectiveness of arthritis induction after subcutaneous CFA injection into the hind paw of outbred guinea pigs and a reproducible development of AIA.

It was shown that injection of arthritis-inducing CFA results in the development of a granulomatous inflammation of the soft tissues of the paw on the injection side, which involves capsules of large and small joints. The active phase of the inflammation in the periarticular tissues remits by day 42 of the experiment. Sclerosis signs develop in 28 days, and further development leads to encapsulation of the inflammation lesion. At the same time, the pathological process in the joint itself, which leads to deep destruction of the articular cartilage and underlying bone, does not stop at the end of the observation period (56 days). This may be indicative of a transformation of arthritis into chronic form. Despite a rather pronounced inflammation in the periarticular tissues, the pathological processes involve not all hind limb joints. The frequency and severity of the damage to the joint clearly depends on the administered dose. In group 200, where the inducing dose was the highest, the most severe disease joint was observed.

In this model, there are fibrin deposits on the synovial surface, leukocyte infiltration of the synovium and surrounding tissue, a productive response in the form of a proliferation of granulation tissue and emergence of angiomatosis areas characteristic of the acute phase of RA. A cellular response in the form of an increased number of plasma cells and formation of follicle-like lymphoid infiltrates, erosive lesions of the cartilage articular surface, often accompanied by deep destruction of large areas of the latter, and epiphyseal lesions, complements the picture of the systemic disease.

It is known that even the use of linear mice and rats usually does not result in the induction of experimental arthritis in all tested animals [1, 2]. This reduces the informative value of the data obtained during pre-clinical trials of new RA treatments. Therefore, the search for well reproducible modes of induced arthritis remains an important area of research [2, 11, 12]. We quantified AIA reproducibility in group 100 guinea pigs. It was shown (*Table*) that injection of CFA at a dose of 100 μl causes significant damage to joint tissue (injection side), which indicates a good reproducibility of arthritis induction in the used RA model and its potential in evaluating antiarthritic drugs and their dosage regimens. It should be noted that, in 28 days, there were no pathological changes in the right (untreated) hind paw or they were much less pronounced than in the left (treated) paw of all group 100 guinea pigs, except for animal number 12 *(Table)*.

**Table:** Assessment of pathological changes in the hind limb tissues of guinea pigs caused by the injection of 100 μl of CFA (see the text for details)

Animal No	On the side of CFA injection (left paw)				Intact (right paw)			
	Inflammation	Synovitis	Cartilage erosion	Bone destruction	Inflammation	Synovitis	Cartilage erosion	Bone destruction
1	3	2	2	0	0	0	0	0
2	2	1	1	0	1	0	0	0
3	2	2	3	0	0	0	0	0
4	3	1	2	1	0	0	0	0
5	2	2	1	0	1	1	0	0
6	2	2	2	0	1	0	0	0
7	3	2	2	0	0	1	0	0
8	2	2	1	0	1	0	0	0
9	1	2	1	0	0	0	0	0
10	2	1	1	0	0	0	0	0
11	2	2	2	1	1	1	1	0
12	1	1	1	0	1	1	3	0
13	2	2	1	0	1	1	0	0
14	1	2	1	0	0	0	0	0

Apparently, the degenerative processes in the underlying bone tissue of group 100 animals did not have enough time to develop to a full extent by that time (28 days), so that the severity of the inflammatory process, which is quite pronounced in the joint tissues, did not always correlate with the damage to the subchondral bone. However, in a model which is used to assess the effectiveness of potential drugs, this is rather an advantage than a drawback, since the far-gone destructive process in the joint was more drug-refractory and thus complicated the assessment of the treatment results.

## CONCLUSION

We have suggested a laboratory model of RA based on a subcutaneous CFA injection to outbred guinea pigs. A high effectiveness and reproducibility of AIA in these animals was shown. The developed model would facilitate pre-clinical testing of antiarthritic drugs.

## Abbreviations

AIA: adjuvant-induced arthritis
RA: rheumatoid arthritis
CFA: complete Freund’s adjuvant
